# Grouting slurry diffusion range based on active heating fiber optics monitoring

**DOI:** 10.1038/s41598-022-22076-5

**Published:** 2022-11-10

**Authors:** Lei Zhu, Wenzhe Gu, Fengqi Qiu, Yibo Ouyang

**Affiliations:** 1 China Coal Energy Research Institute Co., Ltd., Xi’an, 710054 China; 2 School of Energy and Mining Engineering, China University of Mining and Technology (Beijing), Beijing, 100083 China; 3 School of Energy, Xi’an University of Science and Technology, Xi’an, 710054 China

**Keywords:** Civil engineering, Energy infrastructure

## Abstract

To quantify the diffusion range of slurry in grouting engineering, an active heating optical fiber (AHFO) monitoring method is proposed. The AHFO is arranged on the coal seam floor. The temperature is taken as the monitoring parameter to monitor the diffusion range and state of slurry in the injected medium. Considering the time-varying characteristics of slurry rheological parameters, the theoretical calculation formula of spherical diffusion radius based on the power-law fluid is deduced. The relationship between the void ratio and grouting diffusion radius is discussed. Considering the influence of the seepage effect, the "water cement ratio change matrix" in the process of mud seepage is derived, and the influence of the space–time change of the slurry water cement ratio on the temperature gradient of the injected medium is studied. According to the factors affecting grouting diffusion, four groups of small three-dimensional simulation tests and one large three-dimensional grouting test are designed to verify the feasibility of the proposed method. The results show that the relative error of the AHFO monitoring radius is between 3.00 and 14.67%, based on the actual diffusion radius. In the large-scale three-dimensional grouting test, the data from AHFO is used to generate the two-dimensional surface of the grouting diffusion form, and the grouting diffusion range is asymmetric oval. Compared with the theoretical calculation results, the maximum relative error of grouting diffusion radius is 9.6%, and AHFO shows prediction accuracy. With the decrease in the water-cement ratio of slurry, the temperature gradient of the injection medium monitored by AHFO increases, showing an obvious space–time effect. The application of AHFO in the grouting simulation test can effectively analyze slurry diffusion in the injected medium.

## Introduction

A large amount of coal gangue will be produced in coal mine construction and production, resulting in significant environmental problems^1^. After coal mining, a new crushing space is formed underground. Pipeline slurry crushing space-filling technology provides an economical and effective way for coal gangue filling mining. By making full use of the collapse and crushing space in the mined-out area after mining, the gangue is transported to the crushing space in the form of slurry through crushing and grinding to achieve the purpose of efficient disposal of gangue and stability of the mined-out area^2^. Therefore, the study of grouting diffusion range and diffusion form can provide a research basis for the reasonable filling and disposal of coal gangue.

Grouting technology has been widely used in mining, metallurgy, hydropower, railway, construction, petrochemical, and some military projects^3–5^. There are many factors affecting the rapid development of grouting technology, such as grouting materials, grouting equipment, construction personnel quality, etc. However, among many factors, the experimental research based on grouting visualization is the key factor for successfully applying grouting diffusion mechanism theory and grouting technology in practical engineering. So far, people have assumed and simplified some limited known conditions, such as geological conditions and construction process parameters, and studied some grouting diffusion mechanisms, including spherical diffusion theory, cylindrical diffusion theory, casing valve diffusion theory, and so on^6^. There are many problems in applying these theories in grouting engineering practice, which is easy to cause problems such as insufficient grouting and unreasonable selection of grouting hole spacing, resulting in substandard engineering quality and low economic benefits.

Many researchers have studied the range of grouting diffusion. Bezuijen et al.^7^ conducted on-site monitoring of grouting behind a tunnel wall. The results show that when the excavation stops, the buoyancy of the grouting fluid begins to have an impact. At the same time, with the hardening of the slurry, the buoyancy will be reduced, and the gradient of horizontal pressure will be less than that of water pressure. With the development of numerical simulation technology, Ezzeldine et al.^8^ use the finite element program PI-SA to simulate the influence of shield propulsion, lining assembly, and grouting behind the wall on the foundation deformation. However, more research on grouting diffusion range is based on the theoretical formula. G. Lombardi et al.^9^ deduced Bingham type slurry diffusion distance formula for smooth horizontal equal thickness cracks, and Bolisetti et al.^10^ studied grouting fluid viscosity and shear effects stress on grouting effect considering the influence of seepage effect on grouting effect. Bouchelaghem et al.^11^ studied the fluid–solid coupling phenomenon and filtration effect of saturated porous media in the process of being injected into the miscible slurry and established the corresponding theoretical model. Saada et al.^12^ respectively discussed the influencing factors and theoretical models of the infiltration effect of cement slurry in the process of infiltration grouting. Kim et al.^13^ proposed an evaluation method for the groutability of cement slurry in porous media. The development and popularization of artificial neural networks have greatly improved the limitation of theoretical calculations formula and improved people's understanding of grouting technology and theory. Tekin et al.^14^ established an artificial neural network model that can be used to estimate the grouting effect considering the influence of slurry water-cement ratio, the relative density of injected rock and soil mass and grouting pressure. The above studies seldom consider the influence of the percolation effect on the slurry diffusion range. And, because it is difficult to directly observe the grouting diffusion range in the indoor or field test, most scholars use theoretical calculation and numerical simulation to predict the grouting diffusion range. Therefore, realizing the visual monitoring of grouting is essential to promote the development of grouting theory.

Monitoring the diffusion range of the grouting slurry is very important to optimize grouting pressure, grouting time, and grouting hole spacing. However, the review of technical literature shows that the traditional monitoring method of grouting diffusion range is difficult to ensure the density of measurement points, which eventually leads to an inaccurate description of the shape of the grouting diffusion range. The existing nondestructive testing methods use the excitation signal wave to penetrate the structure to be tested and have strict requirements for the testing environment such as surface finish, thickness, material, and buried depth of the structure^15–17^. Therefore, the applicability of these methods is poor. The active heating fiber optics (AHFO) has the advantages of sub-meter spacing, real-time monitoring, and distributed measurement, which shows the potential of monitoring the diffusion range of grouting slurry. Wang et al.^18^ used actively heated fiber Bragg grating sensors to measure soil water content. On this basis, sun et al.^19^ developed an active heating fiber Bragg grating field monitoring system. They applied it to soil moisture monitoring on the loess slope. Liu et al.^20^ proposed a soil dry density measurement method based on the active heating fiber Bragg grating method and applied it to field tests. Unfortunately, there is no report on the application of active heating fiber optics in the diffusion range of grouting slurry in goaf.

Therefore, a new method using active heating fiber optics to monitor the grouting diffusion range in goaf is proposed in this paper. Firstly, the AHFO is arranged in a grid shape, and then the active heating function of the AHFO is used to amplify the temperature difference. Taking the temperature change value as the index, the slurry diffusion range in the whole goaf is monitored. Finally, a large-scale three-dimensional grouting test model is constructed, and the grouting diffusion form and diffusion range under different grouting parameters are monitored by AHFO. At the same time, considering the influence of the seepage effect, the "water cement ratio change matrix" in the process of mud seepage is derived, and the influence of the space–time change of the slurry water cement ratio on the temperature gradient of the injected medium is studied. This study shows that AHFO is helpful in optimizing grouting parameters and has the ability to provide quantitative information about grouting slurry diffusion so as to improve the theoretical model of grouting slurry diffusion.

## Materials and methods

### Active heating fiber optics

Raman DTS temperature measurement system is a method to measure temperature and distance by using the Raman scattering effect and optical time domain reflection technology. When the optical pulse enters the optical fiber, it will produce a beam of Stokes light and an Anti-Stokes light. This scattering is called Raman scattering. According to the Raman scattering theory, under spontaneous Raman scattering, the difference of loss coefficient between Stokes light and Anti-Stokes light propagating in optical fiber is ignored. Two beams of back Raman scattering light will be generated when a particular energy pulse pump light is injected into the fiber. The Stokes scattering light with a wavelength more incredible than the incident light is not affected by temperature, while the Anti-Stokes scattering light with a wavelength less than the incident light has a strong temperature dependence. Therefore, the temperature can be calculated according to the light intensity ratio of Stokes and Anti-Stokes. The distributed temperature measurement along the length of the optical fiber can be realized. The temperature value at any point on the optical fiber can be expressed^21^.

1 $$ T\left( z \right) = \frac{\Delta E/k}{{\ln C - \ln R\left( z \right) + \Delta \alpha z}} $$

where *T*(*z*) is the absolute temperature, *R*(*z*) is the light intensity ratio of Stokes and Anti-Stokes, which is only related to temperature. The external ambient temperature affects the Anti-Stokes light. The ambient temperature can be obtained by comparing the Anti-Stokes light intensity with Stokes light intensity, eliminating the influence of non-temperature factors such as light source signal fluctuation and optical fiber bending. ∆*E* represents the difference of molecular energy states driving Raman scattering. *k* is Boltzmann constant. ∆*α* is the difference between Stokes and Anti-Stokes backscattered light loss coefficients. *z* is the distance from DTS light source. *C* is a calibratable parameter, which is related to the wavelength and frequency of incident light, backscattered Raman light, and photon detector of the instrument.

The sampling interval of the system is 0.1 m, and the multimode carbon fiber heating sensing optical cable with an outer diameter of 4.2 mm is used as the transmission and sensing optical fiber. The carbon fiber heating sensing optical cable comprises an optical cable sheath, carbon fiber heating resistance wire, optical fiber sheath, and optical fiber. The resistance value of carbon fiber heating resistance wire is 18 Ω/m, and the measurement accuracy is 0.2 °C. The AHFO can be regarded as an ideal linear heat source. The heating resistance wire is used as the heat source for heating. The optical fiber is used as the sensing part to measure the temperature. The heat generated by the heating resistance wire is transmitted to the outside of the optical cable.

AH-DTS optical cable can be regarded as an ideal linear heat source. The heating resistance wire is used as the heat source for heating, and the optical fiber is used as the sensing part to measure the temperature. The heat generated by the heating resistance wire is equal to the heat transferred to the outside of the optical cable, plus the heat transferred to the inside of the optical cable. The transient heat conduction problem is simulated according to the heat transfer model theory and boundary conditions. The analytical solution of the temperature change value *T* measured by AH-DTS is^20^:

2 $$ T{ = }T(t) - T_{0} = \frac{Q}{{4\uppi \lambda }}\left[ {lnt + 4\uppi R\lambda + ln\left( {\frac{4K}{{a^{2} c}}} \right)} \right] $$

where *T*(*t*) is the sensor temperature corresponding to the heating time; *T*_*0*_ is the initial soil temperature; *Q* is the heating power per unit length; *λ* Is the thermal conductivity of rock and soil mass; *R* is the contact thermal resistance between optical cable and rock and soil mass per unit length; *K* is the thermal diffusion coefficient of rock and soil mass; *a* is the outer diameter of the sensor; *c* is a constant, generally 1.7811.

After grouting, the water content of rock and soil within the slurry diffusion range increases, and the corresponding thermal conductivity also increases. The temperature inside and outside the grouting diffusion range will be different, so take the temperature change value t measured by AH-DTS as the index to judge the grouting diffusion range.

### Grouting diffusion radius

With the development of grouting diffusion theory, many scholars have proposed that the diffusion of fluid in the injected medium is affected by various effects. From the perspective of the fluid itself, the rheological parameters of fluid have a time effect, and the particle motion of fluid material needs to be considered. The diffusion of fluid in the porous medium is affected by the detour effect and other factors for the injected medium. Therefore, taking power-law fluid as experimental material, a theoretical formula for calculating grouting diffusion radius considering time-varying rheological parameters is established in this paper. When applying this formula, the following assumptions should be put forward:

The basic assumption is to analyze the diffusion process of power flow slurry in segment grouting of shield tunnel. The following assumptions are made in this paper: The injected sand is a uniform and isotropic medium; The grouting fluid is a power-law fluid, ignoring the timeliness of the slurry; Constant pressure and constant speed grouting are adopted, and the slurry enters the grouting body through infiltration and diffusion from the grouting hole; In the process of slurry infiltration, the slurry is hemispherical diffusion without considering the influence of gravity; Ignoring the influence of the curvature of the shield segment, it is considered that the outer surface of the shield segment is plane. The seepage motion equation of time-varying power-law fluid with viscosity is expressed as,

3 $$ V = {\text{e}}^{{\frac{ - kt}{n}}} \left( {\frac{{K_{{\text{e}}} }}{{\mu_{{\text{e}}} }}} \right)^{\frac{1}{n}} \left( { - \frac{{d_{p} }}{{d_{l} }}} \right)^{\frac{1}{n}} $$

where *d*_*l*_ is the assumed length of the microfluidic column, *d*_*p*_ is the pressure difference between the two ends of the microfluidic column, *t* is the grouting time, *k* is the time-varying coefficient, *n* is the rheological index.

Effective permeability *K*_*e*_ and effective viscosity are assumed *μ*_*e*_ can be obtained as follows,

4 $$ \left\{ \begin{gathered} K_{{\text{e}}} = \frac{{\varphi r_{0}^{2} }}{2}\left( {\frac{n}{3n + 1}} \right) \hfill \\ \mu_{{\text{e}}} = c_{0} \left( {\frac{1 + 3n}{{\varphi r_{0} n}}} \right)^{n - 1} \hfill \\ \end{gathered} \right. $$

where *r*_*0*_ is the assumed radius of the circular tube, *φ* is the porosity of the injected medium, and *c*_*0*_ is the initial consistency coefficient.

If the radius of grouting pipe is *l*_*0*_ and the diffusion radius of slurry is *l*_*1*_, the slurry flows out of the grouting hole in the form of constant flow, and its flow rate is *v*_*0*_, then the grouting amount per unit time is:

5 $$ q = AV = 2\pi l^{2} V = v_{0} \pi l_{0}^{{2}} $$

where *A* is the outer surface area of hemispherical slurry at any time, which can be obtained from Eqs. (3) and (5):

6 $$ V = \frac{{v_{0} l_{0}^{2} }}{{2l^{2} }} = \left( {\frac{{K_{{\text{c}}} }}{{u_{{\text{e}}} }}} \right)^{\frac{1}{n}} \left( { - \frac{{{\text{d}}p}}{{{\text{d}}l}}} \right)^{\frac{1}{n}} $$

By using the separated variable integral method in Eq. (6) and substituting into the boundary conditions: when the grouting radius is *l*_*0*_, the slurry pressure is *p*_*0*_, the slurry diffusion radius is *l*_*1*_, and the slurry pressure *p*_*1*_ is equal to the groundwater pressure at that place.

7 $$ {\Delta }p = p_{1} - p_{0} = \frac{{{\text{e}}^{kt} \left( {\frac{{\mu_{{\text{e}}} }}{{K_{{\text{e}}} }}} \right)\left( {\frac{\varphi }{3t}} \right)^{n} }}{1 - 2n}\left( {l_{1}^{1 - 2n} - l_{0}^{1 - 2n} } \right)l_{1}^{3n} $$

where *p*_*1*_ is the grouting pressure, *p*_*0*_ is the groundwater pressure at the grouting point, *l*_*0*_ is the radius of the grouting pipe, and *l*_*1*_ is the grouting diffusion radius at the grouting time *t*.

The relationship between flow rate and flow is:

8 $$ Q_{0} = \pi v_{0} l_{0}^{2} t = \frac{2}{3}\phi \pi l_{0}^{3} $$

Substitute into Eq. (7), so that the diffusion radius *l*_*1*_ of the slurry is:

9 $$ l_{{1}} = l_{0} \sqrt[{2n}]{{\frac{{l_{{1}} }}{{l_{0} }} + \frac{{{\Delta }p(2n - 1)}}{{l_{0} l_{{1}}^{n} }}\frac{{K_{{\text{e}}} }}{{u_{{\text{e}}} }}\left( {\frac{3t}{\phi }} \right)^{{\text{n}}} }} $$

### Analysis of influence mechanism of space–time effect of slurry flow percolation

The percolation effect refers to that in the process of slurry infiltration, due to the filtration of porous media, the slurry particles deviate from their trajectory and constantly stagnate, precipitate, adsorb and deposit. And then occupy space to reduce the pore opening, change the permeability of porous media, and finally block the permeability channel, causing the change of slurry water-cement ratio, which has an obvious space–time effect^22^. Now, based on Darcy law, according to the equivalent relationship between seepage flow and pore volume, a theoretical calculation model of seepage considering the time–space effect of seepage is established:

10 $$ dr_{1} dQ = \left( {H - h_{0} } \right)ak\left( {r_{1} ,t} \right)dt \, $$

where *H* is the grouting pressure head, *h*_*0*_ is the pore pressure head, *r*_*1*_ is the grouting radius, *a* is the sectional area of the grouting pipe, and *k*(*r*_*1*_,*t*) is the function of the seepage coefficient of the slurry varying with the grouting time and diffusion distance.

11 $$ k\left( {r_{1} ,t} \right) = \frac{f(t)}{{f\left( {r_{1} } \right)}} $$

Through integration, there is grouting volume Q.

12 $$ \left\{ \begin{gathered} Q = \frac{{\left( {H - h_{0} } \right)a\int_{0}^{t} f (t){\text{d}}t}}{{\int_{{r_{0} }}^{{r_{1} }} f \left( {r_{1} } \right){\text{d}}r_{1} }} = a\left( {r_{1} - r_{0} } \right) \cdot n \hfill \\ \frac{{\int_{0}^{t} f (t){\text{d}}t}}{{\int_{{r_{0} }}^{{r_{1} }} f \left( {r_{1} } \right){\text{d}}r_{1} }} = \frac{{\left( {r_{1} - r_{0} } \right) \cdot n}}{{H - h_{0} }} \hfill \\ \end{gathered} \right. $$

where *r*_*0*_ is the radius of the grouting pipe, *r*_*0*_ ≪ *r*_*1*_.

From Eq. (12), it can be seen that if the expressions of functions *f*(*t*) and *f*(*r*_1_) are known, they can be solved, and these two expressions can only be obtained through a large number of indoor test analyses. Combining Eqs. (11) and (12), obtaining *k*(*r*_*1*_,*t*) is equivalent to obtaining the seepage coefficient *K*(*r*_*1*_,*t*) and porosity *n*(*r*_*1*_,*t*) of rock and soil mass that is

13 $$ K\left( {r_{1} ,t} \right) = 2\left( {\frac{{n\left( {r_{1} ,t} \right)}}{{1 - n\left( {r_{1} ,t} \right)}}} \right)^{2} d_{10}^{2} $$

Due to the influence of the seepage effect, the seepage coefficient of rock and soil mass changes with time, mainly reflected in the change of porosity, as shown in the following equation:

14 $$ \left\{ {\begin{array}{*{20}l} {\sigma = \beta \alpha \omega tV} \hfill \\ {n(t) = n - \beta \alpha \omega t} \hfill \\ \end{array} } \right. $$

where *σ* is the volume of slurry particles percolated in *t* time, *β* is the expansion coefficient of particles, generally taken as 2.0–3.0. *α* is the percolation coefficient of the slurry, and its value is related to the filtration rate and filtration coefficient, *ω* is the mass fraction of particles in the slurry, *V* is the total volume of rock and soil mass, *n*(*t*) is the real-time porosity of rock and soil mass.

In addition, the water-cement ratio at the near end of the grouting hole is small, and the seepage coefficient is small, which plays a key role in the seepage of the slurry, especially in determining the time when it is difficult to inject the slurry. The judgment calculation equation of grout injectability considers the percolation effect. As shown in the following Eq. (15):

15 $$ N_{{\text{g}}} = \frac{{1.25(n - \beta \alpha \omega t)d_{10} }}{{D_{95} }} $$

where *N*_*g*_ is the injectability parameter, *D*_95_ is the particle size corresponding to the cumulative mass fraction of particles in the slurry less than 95%. *n* is the porosity of rock and soil mass, and *d*_*10*_ is the effective particle size of rock and soil particles.

The increased slurry particle precipitation directly leads to decreased porosity and seepage coefficient. When the pore volume is certain, the change in slurry particle precipitation quantity represents the change in the water-cement ratio. Therefore, the slurry water-cement ratio change is an important embodiment of the percolation effect. Now the water-cement ratio is taken as the research object to analyze its change process and characteristics. Since the slurry infiltration is a step-by-step process, for the convenience of expression, the matrix form is used to describe and establish the "water cement ratio change matrix" of the slurry infiltration process, as shown in the following Eq. (16).

16 $$ {\mathbf{R}}_{ij} = \left[ {\begin{array}{*{20}c} {r_{11} } & \ldots & \ldots & \ldots & \ldots \\ {r_{11} - \Delta r_{21} } & {r_{11} + \Delta r_{11} } & \ldots & \ldots & \ldots \\ {r_{11} - \Delta r_{21} - \Delta r_{31} } & {r_{11} + \Delta r_{11} - \Delta r_{32} } & {r_{11} + \Delta r_{11} + \Delta r_{22} } & \ldots & \ldots \\ {r_{11} - \Delta r_{21} - \Delta r_{31} - \Delta r_{41} } & {r_{11} + \Delta r_{11} - \Delta r_{32} - \Delta r_{42} } & {r_{11} + \Delta r_{11} + \Delta r_{22} - \Delta r_{43} } & {r_{11} + \Delta r_{11} + \Delta r_{22} + \Delta r_{33} } & \ldots \\ \ldots & \ldots & \ldots & \ldots & \ldots \\ \end{array} } \right] $$

where *i* and *j* are the unit length of grouting seepage distance and the unit length of the injected medium, *i* ≥ 1, *j* ≥ 1. It can be seen from the above formula that *r*_*11*_ is the initial water-cement ratio of the slurry, and *r*_*i1*_ ≤ *r*_*11*_, *r*_*ij*_ ≤ *r*_*i (j*+*1)*_ ≤ *r*_*i (j*+*2)*_ ≤ … ≤ *r*_*i (j*+*m)*_ ≤ …, *m* ≥ 1. The water cement ratio of the grout increases along the seepage direction after the grout passes through the percolation. The farther away from the grouting pipe, the greater the water-cement ratio of the grout. It is especially pointed out that the water-cement ratio near the grouting mouth is smaller than the initial water-cement ratio.

The percolation effect has an obvious space–time effect, which is not only related to percolation time but also related to diffusion distance. In the direction parallel to the infiltration direction, the amount of slurry particle precipitation increases with time, and the porosity gradually decreases with the diffusion distance. As the slurry particles fill the porosity of the injected medium to different degrees, the porosity changes differently, and the corresponding thermal conductivity will change. So the temperature reduction value monitored by AHFO will be different and decrease with the increased distance from the grouting pipe. It is consistent with the analysis of test results in subsequent chapters.

**Figure 1:**
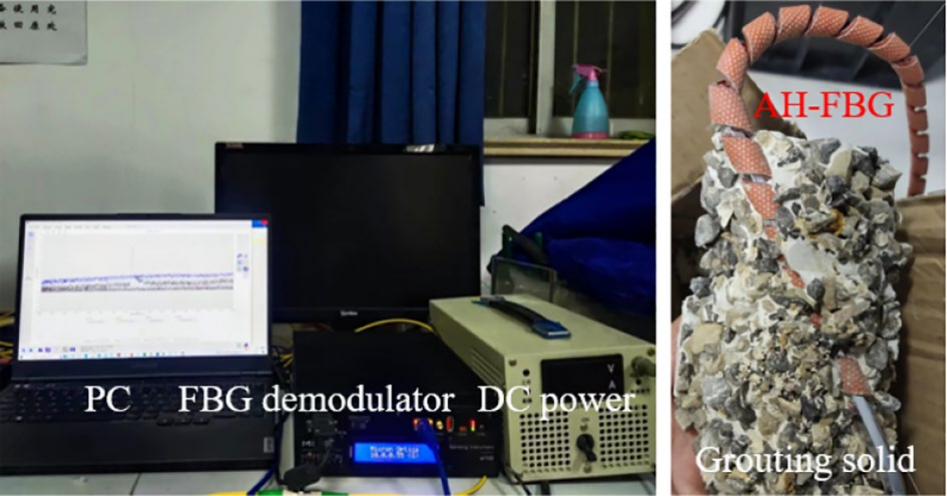
Monitoring system.

To simulate the change of temperature drop of the injected medium under the influence of the percolation effect, the gravel pile infiltration grouting (grout with different water-cement ratio) measurement test was carried out indoors. The test device is shown in Fig. 1. The self-developed actively heated fiber Bragg grating (AH-FBG) sensor comprises a metal bar, a spiral resistance wire and FBG. The diameter of the metal bar is 0.8 mm, the effective heating resistance is 8.0 Ω, and the sensing length is 10 cm. The test uses gravel with a uniform particle size of 1 cm. The size of 20 × 20 × 15 cm is selected for the test plexiglass transparent box, and place AH-FBG sensor in it. Since the slurry injection position is close to the AH-FBG sensor, the influence of the percolation effect can be ignored. After the gravel pavement is completed, the sensors are connected to the adjustable DC power supply and FBG demodulator for heating and collecting the central wavelength data of the sensor. After heating to 60 °C at a constant 35 W/m power, the power is cut off. The wavelength data in the cooling process is recorded after injecting slurry with a water-cement ratio of 0.5–0.9, respectively and recorded in the cooling process.

**Figure 2:**
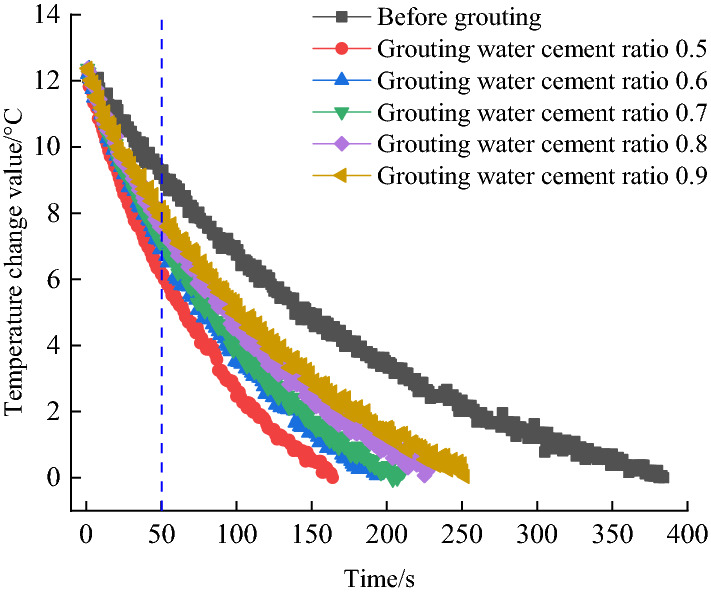
Temperature measurement results.

From the wavelength data recorded by FBG, the gravel pile temperature drops time history curve before and after grouting with different water-cement ratios can be obtained. The right side of Fig. 1 shows the stone body after grouting and the AH-FBG sensor. Intercept the temperature change data in the range of 0–12 °C for analysis, as shown in Fig. 2. When the temperature drop time is 50 s, the temperature drop of gravel before grouting is the lowest. The temperature drop of gravel after grouting with a water cement ratio of 0.5 is the largest. The temperature of the sample decreases with the increase of time, but the duration of temperature reduction and the gradient of temperature drop are different. The main reason for this result is that the average thermal conductivity of mineral particles is about 2.9 W/(m K), while the thermal conductivity of water and gas is 0.6 W/(m K) and 0.024 W/(m K) respectively. With the decrease of the water-cement ratio of the slurry, the number of soil particles in the unit volume increases, the porosity decreases, the air is compressed and discharged, and the particle contact area increases. With the increase of specific heat capacity and thermal conductivity, the temperature drop gradient increases, and the temperature drop time decreases. This also explains that AHFO monitors different temperature drops in subsequent experiments.

## Confirmatory tests

### Test equipment and materials

**Figure 3:**
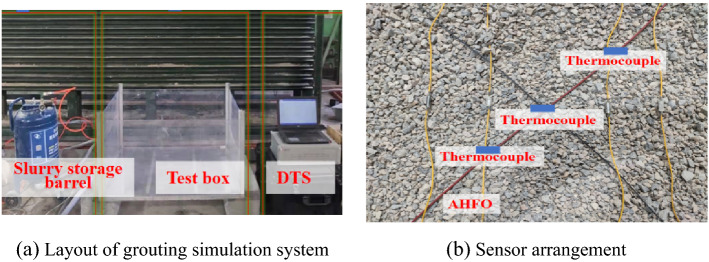
Grouting simulation system.

The grouting simulation system assembled indoors is used for the confirmatory test. The system comprises three parts: pressure supply system, test box system, and monitoring system, as shown in Fig. 3a. The pressure supply system is composed of an air compressor, slurry storage barrel, pressure gauge, pressure regulating valve, grouting pipeline, electronic scale, etc., which can provide stable pressure. The test box is composed of a removable acrylic box, which can accommodate a large diffusion radius. The radius of the stone body can be observed when it is disassembled. The monitoring system is the active heating fiber optics connected DTS distributed optical fiber thermometer and thermocouple thermometer, as shown in Fig. 3b. The temperature signal can be measured directly when the thermocouple is embedded in the measured object. Nickel chromium-nickel silicon thermocouple is used in this test to measure the temperature in the range of 0–1000 °C, with good linearity and measurement accuracy^23,24^. The DTS system used in this test has a spatial resolution of 0.3 m, a sampling interval of 0.1 m and a temperature resolution of 0.1 °C.

Three groups of ordinary gravels with different particle sizes and uniform distribution are selected as the injected medium. Its basic physical properties are shown in Table 1. The grouting material is ordinary portland cement with the commonly used grade of 32.5. Cement slurry with different water-cement ratios can be prepared as power-law fluid and has good injectability. The rheological parameters of slurry with initial temperature were measured by viscometer rheometer. The basic rheological parameters measured are shown in Table 2.

**Table 1:** Relevant parameters of gravel with different particle sizes.

Material number	Particle size (mm)	Permeability coefficient (cm·s^−1^)	Porosity (%)
Material 1	2–5	0.17	29.64
Material 2	5–10	1.36	36.49
Material 3	10–15	7.54	41.73

**Table 2:** Rheological properties of slurry with different water cement ratio.

Water cement ratio	Initial consistency coefficient c_0_ (Pa·sn)	Time varying coefficient k	Rheological index *n*
0.5	4.4685	0.0835	0.2437
0.6	1.2118	0.0673	0.4314
0.7	0.1947	0.0539	0.7439
1.0	0.1131	0.0324	0.9127

### Test design

To verify the feasibility and accuracy of active heating optical fiber monitoring the grouting diffusion range, four experiments were designed to verify the accuracy of active heating optical fiber monitoring the grouting diffusion range under different grouting diffusion ranges. The design test scheme is shown in Table 3.

**Table 3:** Test design scheme.

Test number	Water cement ratio	Grouting pressure (MPa)	Permeability coefficient (cm·s^−1^)	Grouting time (s)
S1	0.5	0.5	7.54	120
S2	0.6	0.4	7.54	60
S3	0.6	0.5	0.17	90
S4	0.7	0.5	1.36	60

Select ordinary gravel, screen the particle sizes of 2–5, 5–10 and 10–15 mm respectively, dry the gravel after cleaning, and measure its basic physical parameters, permeability coefficient and porosity. As shown in Fig. 3, assemble the grouting simulation system, connect the grouting pipeline, load the slurry into the slurry storage barrel in advance, and then adjust the outlet pressure of the air compressor with the pressure regulating valve to ensure that the pressure meets the test design requirements. Fill half of the gravel into the test chamber, lay the optical fiber sensing monitoring line according to the test design, and continue to fill the gravel after the laying. The test is carried out in the room with relatively stable temperature. Slurry with a certain temperature needs to be prepared to meet the requirements of temperature difference with the indoor temperature. The slurry with preparation shall be mixed and poured into the slurry storage barrel. Grouting shall be started immediately to prevent slurry sedimentation. At the same time, the optical fiber monitoring equipment shall be opened to start recording. Grouting shall be timed according to the pressure debugged in advance. The grouting pump shall be shut down after reaching the design grouting time. End grouting, after stopping grouting, continue monitoring with optical fiber monitoring instrument for a certain time to ensure the end of slurry flow, and make time point records. After the slurry solidifies in the gravel, disassemble the test chamber, take out the stone body and record the diffusion. The time difference between mud injection and temperature distribution measurement is 130 s, 70 s, 100 s and 70 s respectively.

### Test results and error analysis

**Figure 4:**
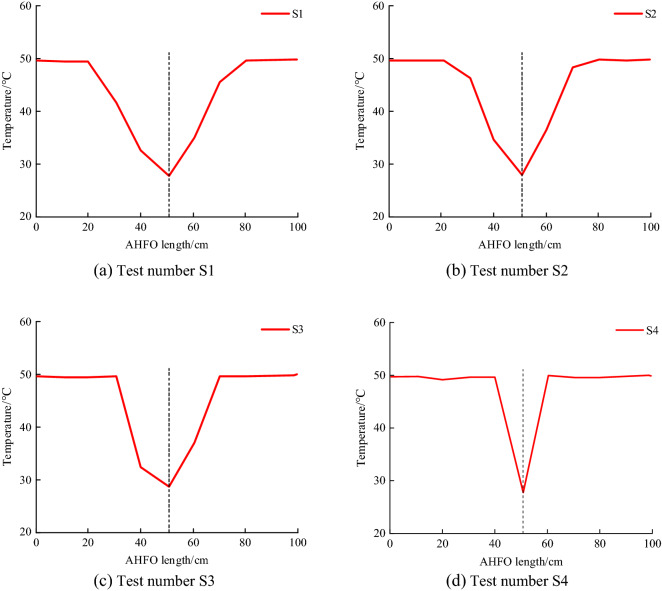
AHFO monitoring results.

AHFO monitors the temperature change caused by the flow of cement slurry in gravel medium. The temperature variation along the optical fiber is shown in Fig. 4.

By monitoring the temperature change along with the optical fiber through AHFO, it can be concluded that the curve is in the shape of a wave crest, and the waveform is similar, which has a certain regularity. Taking the position of the grouting pipe as the center, take 50 cm in front and back along the line, and the abscissa about 50 cm indicates the position of the grouting pipe. The maximum wave peak temperature here is when the slurry is just injected into the medium. The downward trend of the curves on both sides of the grouting pipe is consistent, proving that the temperature decreases continuously during the diffusion process. The temperature difference monitored by the optical fiber proves the flow of the slurry. There is no obvious temperature difference outside the optical fiber at this critical point, so it is judged that the slurry diffusion is over. The ambient temperature weakly affects the temperature at which the slurry does not diffuse to the area. Therefore, it can be determined that the distance between the two bottom angles of the wave crest is the diffusion diameter. It can be seen that the distance between both sides of the grouting pipe is not completely equal, which proves that the plane diffusion shape of the grouting pipe bottom is not regular circular, so the overall shape is not completely spherical. Still, it can be judged that the slurry diffusion in one direction along the optical fiber is good.

Figure 4a and d are different in shape, mainly because the water cement ratio of the slurry in test S1 is smaller, the grouting time is longer, and the permeability coefficient of the injected medium is larger than that in test S2, so the slurry diffusion range is larger than that in test S2. The width of the concave peak of the temperature curve in Fig. 4d is smaller than that in Fig. 4a. The water cement ratio of grout, grouting pressure, permeability coefficient of injected medium and grouting time will all affect the grouting diffusion range, so the shape of temperature curve in Fig. 4 is different. Due to the model size, the number of sampling points in this test is small, so the temperature drop in Fig. 4 is not linear with time. However, affected by the percolation effect of slurry diffusion, the temperature drop data monitored by AHFO varies with the distance from the grouting pipe. As a whole, the farther from the grouting pipe, the smaller the temperature drop data monitored by AHFO.

The optical fiber monitoring results show that when the injected medium cannot be visualized, the optical fiber is placed as the signal transmission and sensing medium. The monitoring effect of temperature is good. Among them, AHFO can directly monitor the slurry diffusion radius of the plane where the grouting pipe bottom is located and can reflect the diffusion effect in a certain direction along with the optical fiber.

The radius value of the grouting diffusion range calculated according to the theoretical formula, the actual stone body radius value measured in the simulation test, and the radius value of the grouting diffusion range monitored by AHFO are compared as shown in Table 4. The actual radius in Table 4 refers to the radius of the stone body.

**Table 4:** Comparison of monitoring results.

Test number	Actual radius (cm)	Theoretical radius (cm)	DTS monitoring radius (cm)	Theoretical and practical errors (%)	DTS monitoring radius and theoretical error (%)	DTS monitoring radius and actual radius error (%)
S1	25.60	35.04	30.00	26.94	16.80	14.67
S2	19.40	27.37	20.00	29.12	36.85	3.00
S3	17.90	20.58	20.00	13.02	2.90	10.50
S4	10.50	13.87	10.00	24.30	38.70	5.00

It can be seen from the chart that the error between the theoretical radius and the actual radius is between 13.02 and 29.12%, and the actual radius of the four groups of tests is less than the theoretical radius. The reasons are as follows: ① due to the natural accumulation of crushed gravel, there are unknown differences in the size of local space voids, and the mudflow process may produce a blocking effect. ② The crushed stone does not reach the wholly isotropic and homogeneous state assumed by the theoretical formula. ③ The adhesion between the mud and the injection medium leads to the retention of solid particles at some positions in the mud, thus increasing the flow resistance. Limited by the system accuracy of the DTS demodulation instrument, the error comes from the system error generated in the process of demodulation and modulation of optical fiber signal by the DTS demodulation instrument, resulting in positioning error. At the same time, the mudflow process may also be affected by seepage effect. As a high-precision optical fiber sensing monitoring method, the monitoring radius is more accurate than the theoretical radius, but there are still some errors. The monitoring error of AHFO is 3.00–14.67%.

The measurement accuracy can be improved by: the calibration test of the temperature sensitivity coefficient is carried out through the constant temperature and humidity box to ensure the temperature measurement accuracy of DTS. The influence range of the AHFO sensor will increase with the heating power, heating time and soil thermal conductivity. Therefore, during the test, the heating time should be extended to ensure the stability of the active heating temperature. Taking the temperature drop measured by the AHFO after slurry injection as the index, the grouting diffusion range is determined.

## Large-scale 3D grouting model test

### Model fabrication

To simulate the actual environment of goaf, taking a coal mine as the engineering background, a large-scale three-dimensional similar model is built by using similar physical simulation materials^25,26^. A similar model simulates the goaf left after mining. The goaf is composed of a natural collapsed roof and similar materials. The diffusion of slurry in goaf is studied by grouting pipe in goaf.

**Figure 5:**
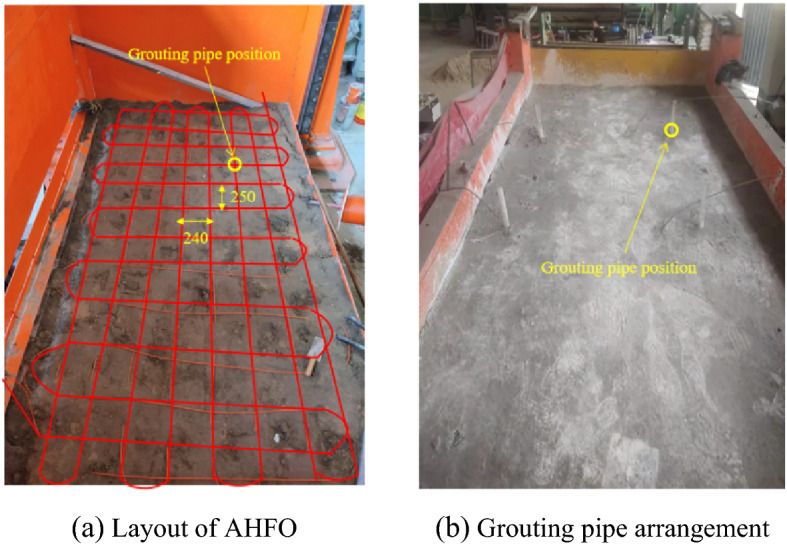
Large-scale 3D grouting model test.

The three-dimensional model is 3600 mm long, 2000 mm wide, and 2030 mm high. At the initial model construction stage, carbon fiber heating optical fiber is laid on the bottom plate, with a total length of about 50 m. According to the theoretical calculation in "Grouting diffusion radius" section, the minimum radius of the grouting diffusion range is 34.64 cm. Therefore, the optical fibers are arranged in a grid shape with a transverse interval of 24 cm and a longitudinal interval of 25 cm. As shown in Fig. 5a, the optical fiber is laid in a cross-grid shape, which realizes the whole area monitoring at the bottom. The diffusion range of the grouting slurry can be accurately tracked and quantified through the optical fiber monitoring data through the two-dimensional spatial positioning algorithm. As shown in Fig. 5b, during subsequent construction, one grouting pipe is embedded at different positions of the coal seam roof.

### Laboratory equipment

**Figure 6:**
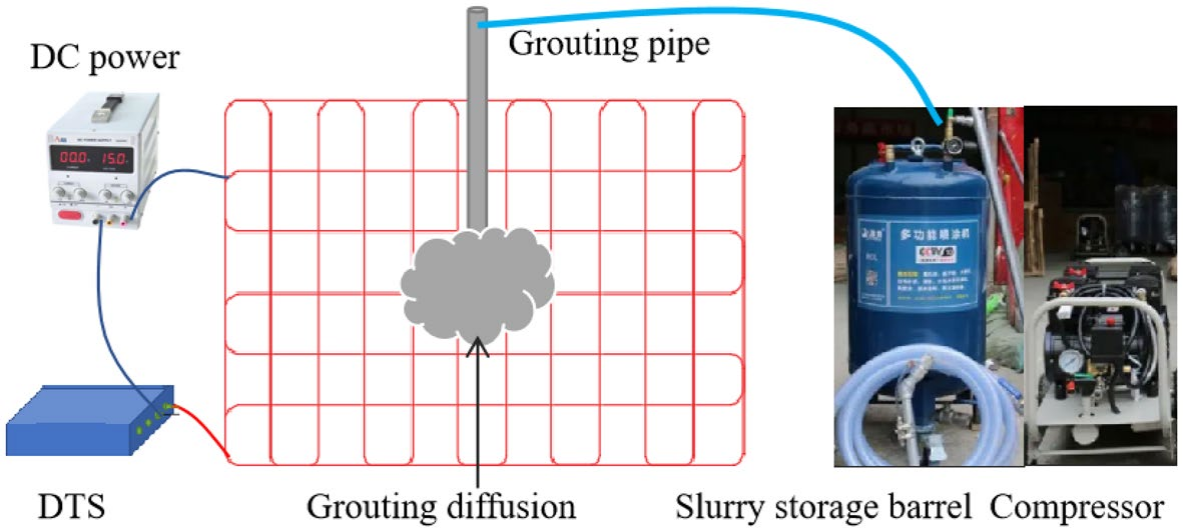
Laboratory equipment.

As shown in Fig. 6, The grouting system is mainly composed of two parts. The pressure supply system is composed of an air compressor, slurry storage barrel, pressure gauge, pressure regulating valve, grouting pipeline, etc., which can provide stable pressure. The monitoring system consists of a carbon fiber heating optical cable, DTS distributed optical fiber thermometer and thermocouple thermometer. Connect the DTS optical cable at the holes on both sides with wires, and connect the other end of the wire to the DC power supply to provide stable voltage. The carbon fiber heating optical cable is connected to the DTS demodulator through jumpers. The thermocouple thermometer can directly measure the temperature. During the measurement, the temperature signal is converted into a potential thermal signal, and the temperature value of the measured object is obtained by signal processing. Nickel chromium-nickel silicon thermocouple is used in this test to measure the temperature in the range of 0–1000 °C, with good linearity and measurement accuracy of 0.1 °C. Used to verify the optical fiber temperature measurement results in the test.

### Experimental process

During the test, the carbon fiber heating optical cable is connected to the DTS system through a jumper to form an optical signal loop to monitor the temperature change of the optical fiber. The carbon fiber heating wire of the carbon fiber heating optical cable is connected to the voltage regulator to set the appropriate heating power. The carbon fiber heating optical fiber is heated to a specific temperature in advance, 50 °C. After grouting, the diffusion range and slurry state in the injected medium are monitored with temperature as the monitoring parameter.

Ordinary portland cement with grade 32.5 is selected for this test. Cement slurry with a water-cement ratio of 1.0, and the flow pattern of cement slurry with the water-cement ratio is Bingham fluid. Cement slurry with different water-cement ratios can be prepared as power-law fluid and has good injectability. The viscometer rheometer measured the rheological parameters of the slurry with initial temperature. The water-cement ratio is 1.00, the initial consistency coefficient is 0.11 PA·S^n^, the time-varying coefficient is 0.03, and the rheological index is 0.91.

The void ratio of the simulated goaf is calculated from the crushing coefficient and expansion coefficient in the test, and its porosity is 43.7%. The water-cement ratio is 1.0, the grouting pressure is 0.3 MPa, the permeability coefficient is 4.69 cm s^-1^, and the grouting time is 120 s. Average temperatures shall be selected for the cement slurry to make the temperature difference between them.

### Experimental results

**Figure 7:**
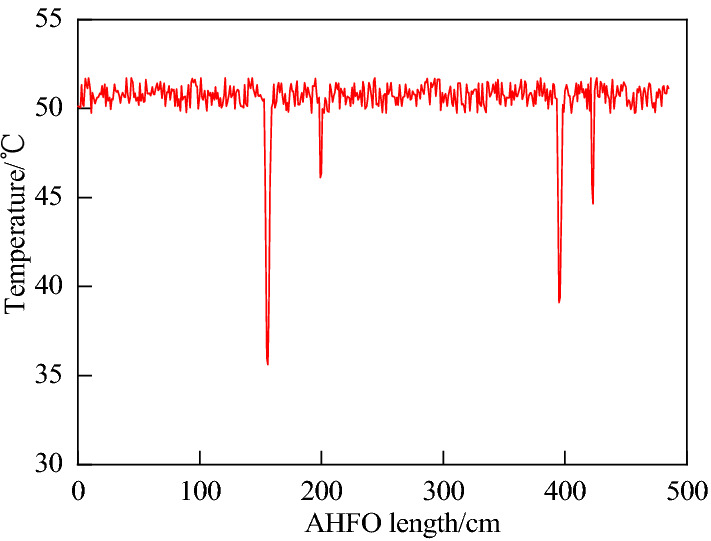
AHFO monitoring results.

After grouting, the slurry flows out from the center of the grouting pipe and spreads around. The water content in the center of the grouting pipe is high, the thermal conductivity is high, and the speed and degree of temperature drop are large. After grouting, the DTS demodulator obtains the temperature along the AHFO line. As shown in Fig. 7, there are four concave valleys in the AHFO monitoring curve. The concave curve represents that the heating optical fiber senses the slurry flow at room temperature. Compared with the confirmatory small-scale model test in "Confirmatory tests" section, the increase in the number of temperature drops is due to the higher spatial resolution obtained by the grid arrangement of optical fibers and the more data obtained. At the same time, the grouting diffusion range is larger than that of the small-scale model test. Therefore, the increase in the number of temperature drops is reflected in Fig. 7. Compared with the confirmatory small-scale model test in "Confirmatory tests" section, the main reason for different temperature drops is the difference between the measurement time and the slurry injection time. For large-scale three-dimensional experiments, different temperature drops on one optical fiber may be because the heat transfer between the optical fibers will affect each other when the grid-shaped optical fibers are arranged. At the same time, it takes a period for the slurry to reach the optical fiber of the bottom plate after being injected by the grouting pipe. Affected by the percolation effect, the water-cement ratio of the slurry in direct contact with the optical fiber will decrease, which may lead to different temperature drops. The difference in temperature drop in the optical fiber monitoring curve in Fig. 7 may also be affected by factors such as the non-uniformity of slurry diffusion and the change of water cement ratio during slurry filtration.

**Figure 8:**
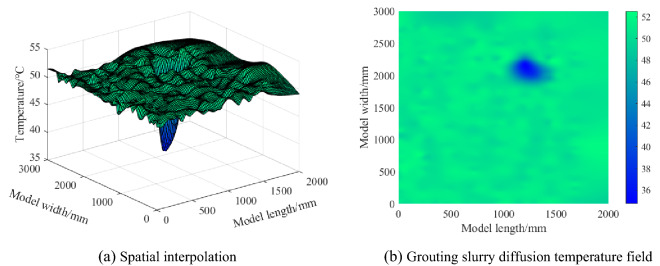
Grouting diffusion range.

Firstly, the monitoring data of the optical fiber is extracted, and then the two-dimensional spatial coordinates of the optical fiber are assigned according to the grid layout. Finally, the Kriging interpolation method is used to make up for the defects of the AHFO grid layout method. The data interpolation is carried out for the range not monitored by the optical fiber to enrich the monitoring data. Finally, the two-dimensional temperature distribution data is formed. As shown in Fig. 8a, a three-dimensional coordinate system is established with the starting position of the optical fiber as the origin, the transverse length of the grid as the X-axis, and the longitudinal length of the grid as the Y-axis, and the monitored temperature as Z-axis. Using the Kriging interpolation method, the temperature field with the grouting pipe as the center is the lowest and gradually radiates around. As shown in Fig. 8b, the grouting diffusion temperature field is shown. At the position of the grouting pipe at the upper right of the model, due to the diffusion of normal temperature slurry, an asymmetric ellipse low-temperature region is formed, which is consistent with the previous research results. The asymmetric ellipse has a major radius of 38 cm and a minor radius of 19 cm. The theoretical diffusion radius calculated is 34.64 cm. Through comparison, it is found that the relative error between the theoretical radius and the major radius monitored by the test is 9.6%.

Different from the assumption that the grouting diffusion range is always simplified to circular in most theoretical calculation models, it is found that the grouting diffusion range of the grouting pipes is asymmetric ellipse in this test. This is caused by the uneven distribution of porosity in the goaf, which is specifically manifested in the preferential flow of slurry to the area with large porosity, resulting in the irregularity of the flow range of slurry. At the same time, the test monitoring results also prove that the porosity of the long axis region in the diffusion range of elliptical grouting is higher than that of the short axis region. It is proved that this monitoring method can monitor the diffusion of slurry, the test accuracy meets the requirements, and the visualization of the diffusion range of slurry can be realized simultaneously.

This shows that the percolation effect affects the slurry, and the water-cement ratio of the slurry increases gradually with the increase of the distance from the grouting pipe. Due to grout deposition, the injected medium's porosity becomes smaller, the original pores are filled with mud particles and water, and the corresponding thermal conductivity increases. Therefore, the temperature distribution map finally monitored by AHFO shows that the temperature drop near the grouting pipe is the largest, and the temperature drop gradually decreases with the increase of the distance from the grouting pipe. The test shows that the seepage effect has an obvious space–time effect, which is related to the seepage time and the diffusion distance. In the direction parallel to the seepage direction, the precipitation amount of slurry particles increases with time, and the porosity decreases with the increase of diffusion distance. With the decrease of the water-cement ratio of the slurry, the number of soil particles in the unit volume increases, the porosity decreases, the air is compressed and discharged, the particle contact area increases, the specific heat capacity increases, and the thermal conductivity increases, leading to the increase of the temperature drop gradient and the decrease of the temperature drop time. This is consistent with the theoretical prediction above.

The economic benefits of the AHFO for field application are mainly reflected in: Due to the inaccessibility of coal mine goaf, it is difficult to monitor the grouting diffusion range in the goaf. Using the AHFO to monitor the grouting diffusion range in the goaf can further optimize the grouting pressure, time, and hole spacing to save cost and achieve the best grouting effect at the same time. In terms of extension, the traditional point measurement layout in the test is difficult, time-consuming, labour-consuming and high cost, but the AHFO realizes distributed measurement, and the relative cost is low. Therefore, applying this innovative means can solve the problems that are difficult to overcome by traditional means and save costs.

## Discussion

There are many reasons for the error between theoretical calculation and monitoring radius. In the grid layout method of the AHFO, its grid size equals the "spatial resolution" of grouting diffusion monitoring. The smaller the grid size, the higher the grouting diffusion range's monitoring accuracy. However, due to the optical loss characteristics of the optical fiber, its bending rate is limited, limiting the monitoring accuracy of the grid layout network formed by an optical fiber. The research results show a balance between the grid size and the monitoring accuracy. The identification and positioning method of the grouting diffusion range is mainly limited by the system accuracy of the DTS demodulation instrument. The error of this mathematical model mainly comes from the system error generated in the process of demodulation and modulation of optical fiber signal by the DTS demodulation instrument, resulting in positioning error. The Kriging interpolation method is essentially introduced to make up for the defects of the grid layout method of the AHFO. The estimated value obtained by the Kriging interpolation method inevitably has a certain error from the actual value. Still, it also makes up for the disadvantage that the grid of the AHFO is not dense enough to a certain extent. The error of Raman DTS measurement is the temperature measurement accuracy, which impacts the identification of the grouting diffusion range. In terms of model making mainly lies in the hydrophilicity of similar materials, temperature and moisture content of similar materials, and the porosity formed after model excavation is also randomly distributed.

Since the above involves many possible error sources such as mathematical interpretation, model making and Raman DTS measurement, the errors shown in the paper are mainly the comparison of the results obtained by using the theoretical calculation formula of grouting diffusion range monitored by the method proposed in the paper and grouting diffusion radius. Although it is difficult to comprehensively analyze and quantify their errors and mutual influence mechanism directly, the accuracy of the proposed method can be verified by comparing the final monitoring results with the theoretical grouting diffusion radius.

Of course, there are some uncertainties in the experiment itself. Firstly, the natural accumulation of collapsed rock blocks and the compaction of overlying strata lead to unknown differences in the size of local space voids. The slurry flow process may produce a blocking effect and dominant path flow effect, so it is reasonable that the grouting diffusion form is an asymmetric ellipse. The rock of the natural collapse of the roof in the goaf is an irregular rock block, resulting in different sizes of internal pores, which do not reach the completely isotropic and uniform state assumed by the theoretical formula. The porosity of the roof strata in the goaf simulated by the test is random and uneven, which is more in line with the actual situation. In the process of slurry flow, gangue particles will move, driven by water flow. Still, they are easy to deposit in the area with small pores and block the pores, leading to the error between theoretical calculation and actual monitoring. Due to the strong water absorption of similar materials, in the process of mudflow, the materials absorb more water, increase the mud viscosity and flow resistance, and can not reach the theoretical diffusion radius. The adhesion between the mud and the injected medium causes the retention of solid particles at some locations, thereby increasing the flow resistance. At the same time, the mudflow process may also be affected by some flow effects. Since the active heating fiber optics monitoring technology takes the temperature change value as the index to monitor the grouting diffusion range, but the slurry is composed of water and gangue particles, the active heating fiber optics monitors the grouting diffusion range, which is the flow range of water in the slurry. In further research, various parameters such as moisture content and thermal conductivity should be used as indicators to accurately monitor the diffusion range of the slurry.

## Conclusions

This paper proposes a monitor method of grouting diffusion range in goaf based on AHFO. This method quantifies the grouting diffusion range by identifying the temperature anomaly in the AHFO temperature profile. Through a large-scale three-dimensional grouting test, the feasibility of this method is verified, and the rapid positioning and identification of the diffusion range of grouting slurry in goaf are realized. AHFO is used to monitor the form and range of grouting diffusion under different grouting parameters. The main conclusions are as follows.

1. Considering the time-varying characteristics of mud rheological parameters, the theoretical calculation formula of spherical diffusion radius of power-law fluid is deduced. Combined with the small-scale three-dimensional simulation test, taking the temperature as the monitoring index, AHFO is used to inverse the grouting diffusion radius. Compared with the measured radius, the relative error of the AHFO monitoring radius is 3.00–14.67%. The results show that AHFO is an effective monitoring method that can effectively monitor the mud diffusion radius.
2. Using a large-scale three-dimensional model test, an optical fiber grid is laid on the coal seam floor to simulate goaf grouting. The temperature change data monitored by AHFO generates the cloud map of the temperature field caused by grouting diffusion. The mud diffusion range can be quantified according to the temperature range. The farther away from the grouting pipe, the smaller the temperature drop.
3. The shape and size of the grouting diffusion range are directly related to the void ratio of goaf. Due to the uneven distribution of pores in the goaf, the grouting diffusion radius in the non-compacted area is large, and the grouting diffusion radius in the compacted area is small. The grouting diffusion shape is finally asymmetric oval, with a major radius of 38 cm and a minor radius of 19 cm. Compared with the theoretical calculation results, the maximum relative error is 9.6%, which shows the accuracy of AHFO monitoring.
4. Through the analysis of the space–time effect mechanism of mud seepage and the grouting test results, it is found that in the direction parallel to the seepage direction, the precipitation amount of slurry particles increases with time, and the porosity decreases with the increase of diffusion distance. With the decrease of the water-cement ratio of the slurry, the number of soil particles in the unit volume increases, the porosity decreases, the specific heat capacity increases, and the thermal conductivity increases, increasing the temperature drop and temperature drop gradient monitored by AHFO.

## Data availability

The datasets used and/or analysed during the current study available from the corresponding author on reasonable request.
